# Enhanced Antifungal Activity of Amphotericin B Bound to Albumin: A “Trojan Horse” Effect of the Protein

**DOI:** 10.1021/acs.jpcb.3c01168

**Published:** 2023-04-18

**Authors:** Ewa Grela, Sylwia Stączek, Monika Nowak, Bozena Pawlikowska-Pawlega, Agnieszka Zdybicka-Barabas, Sebastian Janik, Małgorzata Cytryńska, Wojciech Grudzinski, Wieslaw I. Gruszecki, Rafal Luchowski

**Affiliations:** † Department of Biophysics, Institute of Physics, Faculty of Mathematics, Physics and Informatics, Maria Curie-Sklodowska University, 20-031 Lublin, Poland; ‡ Department of Immunobiology, Faculty of Biology and Biotechnology, Institute of Biological Sciences, Maria Curie-Sklodowska University, 20-033 Lublin, Poland; § Department of Functional Anatomy and Cytobiology, Faculty of Biology and Biotechnology, Institute of Biological Sciences, Maria Curie-Sklodowska University, 20-033 Lublin, Poland

## Abstract

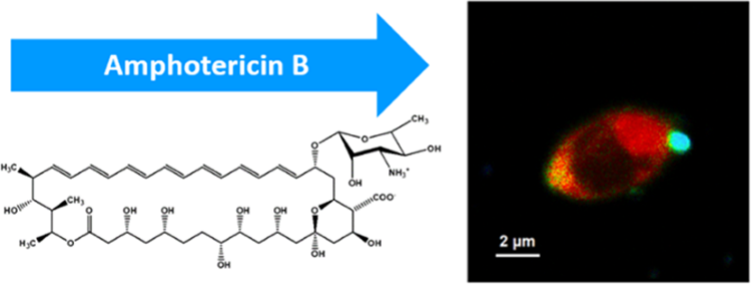

Amphotericin B (AmB) is a life-saving and widely used antifungal antibiotic, but its therapeutic applicability is limited due to severe side effects. Here, we report that the formulation of the drug based on a complex with albumin (BSA) is highly effective against *Candida albicans* at relatively low concentrations, which implies lower toxicity to patients. This was also concluded based on the comparison with antifungal activities of other popular commercial formulations of the drug, such as Fungizone and AmBisome. Several molecular spectroscopy and imaging techniques, e.g., fluorescence lifetime imaging microscopy (FLIM), were applied to understand the phenomenon of enhanced antifungal activity of the AmB–BSA complex. The results show that the drug molecules bound to the protein remain mostly monomeric and are most likely bound in the pocket responsible for the capture of small molecules by this transport protein. The results of molecular imaging of single complex particles indicate that in most cases, the antibiotic–protein stoichiometry is 1:1. All of the analyses of the AmB–BSA system exclude the presence of the antibiotic aggregates potentially toxic to patients. Cell imaging shows that BSA-bound AmB molecules can readily bind to fungal cell membranes, unlike drug molecules present in the aqueous phase, which are effectively retained by the cell wall barrier. The advantages and prospects of pharmacological use of AmB complexed with proteins are discussed.

## Introduction

**Figure 1:**
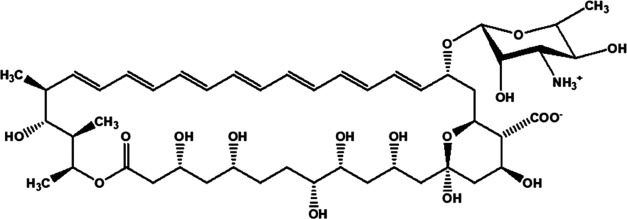
Chemical structure of amphotericin B.

Amphotericin B (AmB, see Figure 1 for the chemical structure), synthesized by *Streptomyces nodosus*, belongs to a class of polyene antibiotics used to treat systemic mycotic infections.^1^ The drug is a life-saving antibiotic and owing to this fact is in use for decades, despite severe side effects.^2^ Research activity of numerous laboratories worldwide is focused on understanding the molecular mechanisms underlying the biological activity of AmB, which can help reduce the toxicity of the drug for patients while maintaining the pharmacological activity against fungi. Among such putative molecular mechanisms of AmB activity, the most frequently reported is the formation of transmembrane pores affecting the physiological ion transport^3−5^ and the activity of the drug molecules leading to interfering with the integrity of biomembranes via disturbance of structural properties of lipid bilayers^6^ and/or sequestration of membrane sterols associated with the formation of extramembranous AmB-sterol bulk structures.^7^ Another direction of the research activity is associated with the elaboration of a pharmacological formula characterized by enhanced antimycotic effectiveness. In this context, several methods for delivering an antibiotic have been proposed, such as mixtures with detergents, liposomes, or hybrid nanoparticles.^2,8^

It is also worth mentioning that the association of AmB with transport proteins, like albumins, allows for the reduction of antibiotic toxicity by prevention of its aggregation, which corresponds with a model where the monomer of AmB is primarily effective in working against fungi. It has been reported that bovine serum albumin (BSA) lowers hemolytic activity and increases critical aggregation concentration of AmB.^9−11^

Research on new formulations of AmB and their interactions with other molecules, which have a significant impact on their pharmacokinetics, is very important in reducing the toxicity of this antibiotic. Here, we present our attempts to find a formulation of AmB with possibly high antifungal activity at relatively low concentrations of the drug, safe for patients. We apply several experimental approaches and techniques to understand both the structure of the effective AmB–protein system and the molecular mechanisms underlying its enhanced antifungal activity. The results show that the activity of AmB bound to albumin relies on the fact that the protein serves as the drug carrier that allows it to pass through the cell wall barrier and integrate with the cell membrane of fungi.

## Experimental Section

### Yeast Strain

The yeast *Candida albicans* (wild-type, a clinical isolate from the human oral cavity; kindly gifted by Prof. A. Kedzia, Department of Oral Microbiology, Medical University of Gdansk, Poland) was cultivated as described previously.^12,13^ The yeast cells in the logarithmic phase of growth were used.

### Materials

Amphotericin B, dimethyl sulfoxide (DMSO), bovine serum albumin, phosphate-buffered saline (PBS), distearoyl phosphatidylglycerol sodium salt, disodium succinate hexahydrate, cholesterol, and sodium deoxycholate were obtained from Merck (Germany). Methanol, chloroform, and sucrose were purchased from POCH (Poland). α-Tocopherol was received from CPAchem (Bulgaria) and hydrogenated soy phosphatidylcholine from Avanti Polar Lipids. Water used in all experiments was purified by a Milli-Q system from Merck Millipore (Germany). Blood was obtained from the Regional Center for Blood Donation and Blood Therapy in Lublin, Poland (consent RCKiK.DN.0262/40/2021). The volunteers gave written consent for the donation of their blood for scientific purposes.

### Amphotericin B Purification

AmB was purified by suspension of 50 mg of antibiotic in a 200 mL mixture of water and chloroform (in a volume ratio of 1:1) and shaking for 30 min. Then, AmB was collected from the interphase of both solvents as described previously.^14^ The described procedure was repeated three times, and then, AmB was dried under nitrogen gas.

### Preparation of Fungizone and AmBisome

All ingredients and the preparation procedure for AmBisome and Fungizone were obtained from Gilead Sciences and Apothecon, respectively. AmBisome ingredients according to Gilead: 1 vial: 50 mg of amphotericin B intercalated into the liposomes membrane consisting of 0.64 mg of α-tocopherol, 52 mg of cholesterol, 84 mg of distearoyl phosphatidylglycerol sodium salt, and 213 mg of hydrogenated soy phosphatidylcholine. Preparation formed by microemulsification in the buffer containing 27 mg of disodium succinate hexahydrate and 900 mg of sucrose in 12 mL of its volume (pH 5–6).

Fungizone: Each vial contained 50 mg of amphotericin B and 41 mg of sodium desoxycholate with 20.2 mg of sodium phosphate as a buffer, which was reconstituted in 10 mL of water. AmB due to its insolubility in water, in this formulation, contained the addition of sodium desoxycholate, which provided a colloidal dispersion of the mixture.

### Complexation of AmB with BSA

The binding of AmB to BSA was performed in a solution of both components. 1.25 μmol BSA was dissolved in 25 mL of PBS buffer (pH 7.4, 10 mM), and then, AmB solution in DMSO (0.5 μmol) was added dropwise into the continuously stirred protein solution. The molecular ratio of BSA/AmB was 5:2. The mixture was incubated with stirring for 1 h at 37 °C. The final DMSO concentration was 1%. To remove AmB aggregates, both attached to BSA and present in the water phase, the sample was centrifuged for 5 min at 36 670*g* (the supernatant was used in the experiments). In all of the experiments, a freshly prepared complex was used.

### Size Analysis of AmB–BSA Complexes

Particle size studies were performed using a Zetasizer Nano ZS analyzer (Malvern, U.K.) at 25 °C.

### Fourier Transform Infrared (FTIR) Analysis of AmB–BSA

Fourier transform infrared-attenuated total reflectance (FTIR-ATR) measurements were recorded on a Nicolet iS50 spectrometer using diamond crystal (Thermo Scientific). The stability of the AmB–BSA complex was tested at different intervals of incubation time at 37 °C. 5% of D_2_O was added into the solution phase of every sample containing AmB, BSA, and AmB–BSA complex, then the samples were partially dried with nitrogen gas by depositing the sample directly onto a diamond reflector. A solvent correction was made based on the D_2_O band beyond the principal bands used for analyses, as tested and applied previously.^15^ Typically, 10 scans were collected, Fourier transform, and averaged for each measurement. Absorption spectra at a resolution of one data point every 4 cm^–1^ were obtained in the region between 4000 and 400 cm^–1^. An increase in the absorption spectra was monitored in the Amide I region. Spectral analyses were performed with Grams software from Galactic Industries.

### Viability Assay

The *C. albicans* survival rate after the treatment with AmB–BSA and AmB was determined using a colony-counting assay, as described in our earlier works.^12,13^ In brief, the log-phase intact *C. albicans* cells (50 μL OD_600_ = 0.02; ∼4.5 × 10^3^ colony-forming units) suspended in 20 mM phosphate buffer, pH 7.4 were centrifuged (6000*g*, 10 min), and then, the buffer was removed and the cells were resuspended in 25 μL of different concentrations of AmB–BSA (final concentrations 0.125–2 μM AmB), AmB (final concentrations 0.125–2 μM), or BSA (final concentrations 2.7–43 μM) dissolved in 1% DMSO. Control cells were incubated with 1% DMSO. All samples were incubated for 0.5 h at 37 °C. Next, serial dilutions of the cells were plated onto solid YPD (1% yeast extract, 2% peptone, 2% dextrose, 1.6% agar) medium, and the grown colonies were counted after 24 h incubation at 37 °C. The number of colony-forming units (CFU) was determined. The control defined the total (100%) survival of *C. albicans* cells in the samples incubated in the presence of 1% DMSO. For comparison, the survival of *C. albicans* after incubation with the antifungal drugs used in the treatment of fungal infections, AmBisome and Fungizone, was analyzed (final concentrations of AmB ranging from 0.125 to 20 μM). The control cells were incubated with a buffer containing disodium succinate hexahydrate and sucrose or PBS for AmBisome or Fungizone, respectively. Antifungal susceptibility to AmB–BSA and AmB was determined as minimum inhibitory concentration (MIC), which is defined as the lowest concentration, which inhibits visible fungal growth after 24 h incubation. The range of final concentrations used for determining the MIC values of AmB–BSA was 0.0125–2 μM and AmB was 0.095–100 μM. *C. albicans* cells taken from solid YPD medium were suspended in liquid YPD medium and grown overnight at 37 °C. Fresh YPD medium was added to the overnight culture and the fungal cells were grown until the log phase. The suspension of log-phase *C. albicans* cells (OD_600_ = 0.1, ∼10^6^ CFU/mL) was prepared and then diluted to obtain 2 × 10^4^ CFU/mL. The MIC values were determined in 96-well plates using a microdilution susceptibility test. The sample final volume in each well was 200 μL and contained 10^4^ CFU. Optical density was measured in a microtiter plate reader (Benchmark Plus, BioRad). Three technical repetitions were performed for each sample.

Additionally, the minimum fungicidal concentrations (MFC) of AmB and AmB–BSA against *C. albicans* were determined. For this purpose, an aliquot of 50 μL of the suspension was taken from a well of the microplate, where no growth (no turbidity) was observed and plated on a YPD agar plate. Subsequently, the plates were incubated at 37 °C for 48 h. The lowest concentration at which ≤1 colony growth was observed was considered the MFC.

### Hemolysis in an Isotonic Solution

Blood samples were drawn from human volunteers by venipuncture. To prevent blood clotting, dry sprayed K2EDTA (dipotassium edetate, ethylenediaminetetraacetic dipotassium) test tubes were used. After washing three times with PBS (154 mM NaCl, 10 mM sodium phosphate, pH 7.4), 50% v/v saline solution from packed red blood cells (RBC) was prepared. Then, 20 μL of erythrocyte suspension was added to 3 mL of isotonic PBS solution in siliconized glass tubes. Three variants of the experiment were performed: samples with 1 μM AmB, samples with 21.5 μM BSA, and samples with the addition of AmB–BSA (1 μM AmB: 21.5 μM BSA). The highest concentration of DMSO was 0.5%. Each experiment was performed in triplicate. After 1 h of incubation at 37 °C, the reaction mixtures were centrifuged at 2000*g* for 5 min. Subsequently, the absorbance of the supernatant was determined at 540 nm. Next, the relative hemolysis was calculated regarding a sample showing 100% hemolysis. Five independent investigations were performed with RBC obtained from different blood donors.

### Steady-State and Time-Resolved Fluorescence and Fluorescence Anisotropy Measurements

For analysis, the log-phase *C. albicans* cells (200 μL of suspension; OD_600_ = 0.02) in 10 times diluted YPD medium were centrifuged (6000*g*, 10 min), and the pellets were washed four times with 20 mM phosphate buffer, pH 7.4 (200 μL), and finally, the yeast cells were suspended in 100 μL of 20 mM phosphate buffer, pH 7.4. Next, 20 μL of appropriate suspension of *C. albicans* cells was applied on a polylysine-coated coverslip and the selected cell was imaged with the FLIM technique. Subsequently, 10 μL of 2 μM AmB–BSA prepared in 1% DMSO was added. Identical measurements were carried out for yeast cells before and after the addition of Fugizone or AmBisome (in both cases the concentration of AmB was 2 μM). The analyses were carried out for no longer than 0.5 h.

Absorption spectra were recorded with a Cary 60 UV–Vis spectrophotometer (Agilent Technologies). The concentration of AmB was determined based on the molar extinction coefficient (121 400 M^–1^ cm^–1^) in the DMSO solution.^16^ In the case of AmB in the protein complex, the concentration of the antibiotic was established based on the molar extinction coefficient, which is 130 000 M^–1^ cm^–1^ and corresponds to 0–0 absorption maximum.^17^

Microscopy data were recorded on a MicroTime 200 confocal system from PicoQuant GmbH (Germany) connected to an Olympus IX71 (Japan) inverted microscope. In all experiments, a water-immersed objective (Olympus NA = 1.2, 60×) was used. The samples were excited with a 405 nm laser working at a 10 MHz repetition rate. The observation was performed using a 50 μm diameter pinhole and optic filters: ZT 405RDC dichroic filter, ZET405 StopLine Notch Filter, and 405 nm long-pass filter (all purchased from Chroma-AHF Analysentechnik, Germany). The fluorescence signal was split into parallel and perpendicular polarized channels. In the case of single-particle measurements carried out for the AmB–BSA complex (concentration of AmB at 10^–10^ M), all measurement settings and parameters were identical except the repetition rate of the laser, which was 20 MHz. The time traces of the intensity of fluorescence emission were collected for 30 s. Fluorescence lifetimes, intensities, and fluorescence anisotropy values were analyzed using the SymPhoTime 64 v. 2.6 software (PiqoQuant GmbH, Germany). Fluorescence emission spectra from *C. albicans* cells were recorded with spectrograph Shamrock 163 attached to the microscopy system. Newton EMCCD DU970P BUF camera (Andor Technology, U.K.) cooled to −50 °C was used as a detection system in these measurements.

Fluorescence anisotropy decays and fluorescence emission measurements of bulk solutions of AmB–BSA complex and AmB were carried out using a FluoTime 300 spectrometer (PicoQuant GmbH, Germany). The maximal absorption of the sample was kept below 0.1. Emission data were obtained by exciting the sample with a 405 nm laser operating with a repetition rate of 20 MHz. A 405 nm long-pass filter from Chroma-AHF Analysentechnik (Germany) was applied. Fluorescence emission intensity decays were recorded in the 430–480 nm wavelength range. Fluorescence anisotropy was calculated according to the formula
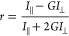
1where *I*_∥_ is the parallel (to the direction of polarization of excitation light) fluorescence intensity, *I*_⊥_ is the perpendicular fluorescence intensity, and *G* is the instrumental correction factor determined before each measurement.

Fluorescence anisotropy kinetics were calculated according to the formulas
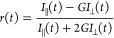
2where *I*_∥_(*t*) is the time-dependent decay of parallel fluorescence emission, *I*_⊥_(*t*) is the time-dependent decay of perpendicular fluorescence emission, and
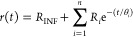
3where *R*_INF_ is the value of anisotropy in infinity, *R*_i_ is an initial anisotropy, and θ_i_ is the rotational correlation time.

Fluorescence emission intensity decays were fitted according to the equation below
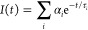
4where α_*i*_ is the relative amplitude of the *i* component and τ_*i*_ is the decay time.

### Scanning Electron Microscopy (SEM) of *C. albicans* Cells

*C. albicans* cells were incubated at 37 °C for 1 and 2 h with AmB, AmBisome, Fungizone, or with AmB–BSA complex at the AmB concentration of 2 μM. The control cells were incubated with PBS, 1% DMSO, or BSA. After gentle centrifuging (2500*g*, at 4 °C), the pellet of the cells was fixed with 4% glutaraldehyde in 0.1 M phosphate buffer pH 7.2 for 2 h at 4 °C. Then, the samples were rinsed with 0.1 M phosphate buffer (pH 7.2). After washing, post-fixation was performed with freshly prepared 1% osmium tetroxide at 4 °C for 2 h. The subsequent rinsing was carried out with the usage of 0.1 M phosphate buffer (pH 7.2). Then, the cells were dehydrated in a series of ethanol gradients: 30, 50, 70, 90, and 100% (each for 10 min). The next step was chemical drying with the application of 98% hexamethyldisilazane (HMDS). Eventually, the specimens were coated with gold in Emitech K550X Sputter Coater. The sample analysis was performed with a TESCAN vega 3 LMU scanning electron microscope (Czech Republic). For every experimental variant, the percentage of altered cells was calculated from at least eight microscopic fields of observed cells. Then, the percentage was averaged.

## Results and Discussion

### Characterization of the AmB–BSA Complex

**Figure 2:**
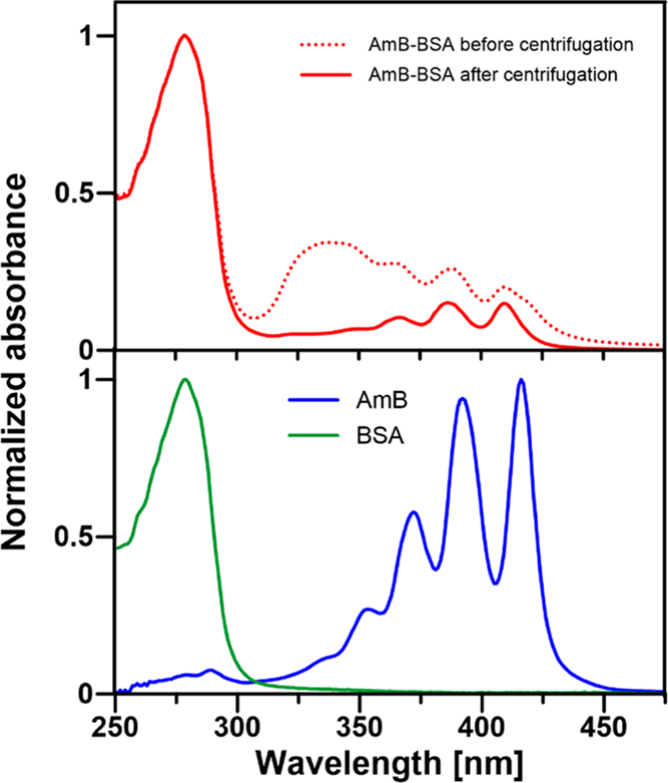
Absorption spectra of the AmB–BSA complex (upper panel) before and after centrifugation aimed at the elimination of antibiotic aggregates either bound or unbound to protein. The lower panel shows the absorption spectra recorded from pure components BSA (in the buffer solution) and AmB (recorded in DMSO). The spectra were normalized at the absorption maxima.

**Figure 3:**
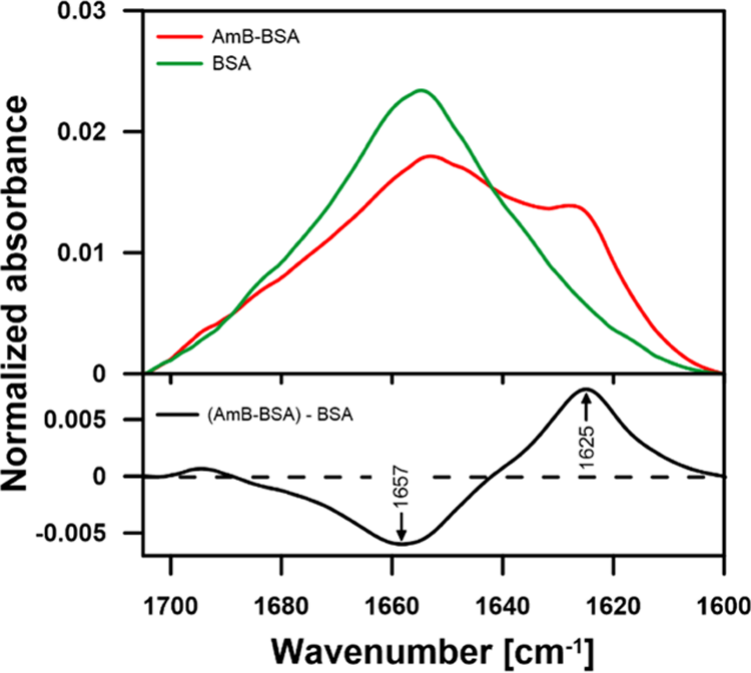
FTIR spectra of BSA and AmB–BSA complex (upper panel). The spectra were normalized to the area under the curve. The lower panel shows the difference spectrum calculated from the spectra presented in the upper panel. See the text for more explanations.

Complexes of AmB formed with bovine serum albumin (AmB–BSA) were characterized with the application of variable molecular spectroscopy techniques. Analysis of the electronic absorption spectra recorded in the UV–Vis region leads to the conclusion that molecules of AmB complexed with BSA remain in a monomeric form (Figure 2). This means that during the centrifugation stage, the aggregated AmB forms giving rise to the broadband between 315 and 355 nm^18^ are effectively eliminated from the prepared samples, regardless of whether they are present in the water phase or are bound to the protein. The particle size analysis shows that both the BSA and AmB–BSA solutions are homogeneous, composed of protein monomers and characterized by a relatively low polydispersity parameter of 0.118 (see Figure S1). The comparison of the concentrations of BSA and AmB in the sample of the AmB–BSA complexes formed, based on the molar extinction coefficients of the drug and the protein and the absorption spectra recorded (Figure 2), leads to the conclusion that statistically, one antibiotic molecule is bound by every 20th protein molecule. IR absorption spectroscopy was additionally applied to address the problem of AmB complexation with protein because such analysis is a tool sensitive to structural modifications of proteins and their molecular organization.^19,20^Figure 3 presents the FTIR absorption spectra recorded from pure BSA and AmB–BSA complex in the Amide I region. Pronounced spectral changes are diagnostic for a certain structural reorganization of the protein upon complexation with the antibiotic. In particular, the appearance of the band centering at 1625 cm^–1^ at the expense of the spectral component centering at 1657 cm^–1^ (see the difference spectrum in the lower panel, Figure 3) can be interpreted in terms of a slight refolding of BSA giving rise to parallel β-structures at the expense of α-helix forms.^19,20^ The fact that such a spectral effect is reversible after prolonged incubation (Figure S2) suggests that this molecular rearrangement is most likely related to the transient binding of single antibiotic molecules to the binding pocket of this transport protein. The results of the FTIR analyses show also that the samples of the AmB–BSA complexes were found to be relatively stable (over periods ranging for 2 h, see Figure S2).

**Figure 4:**
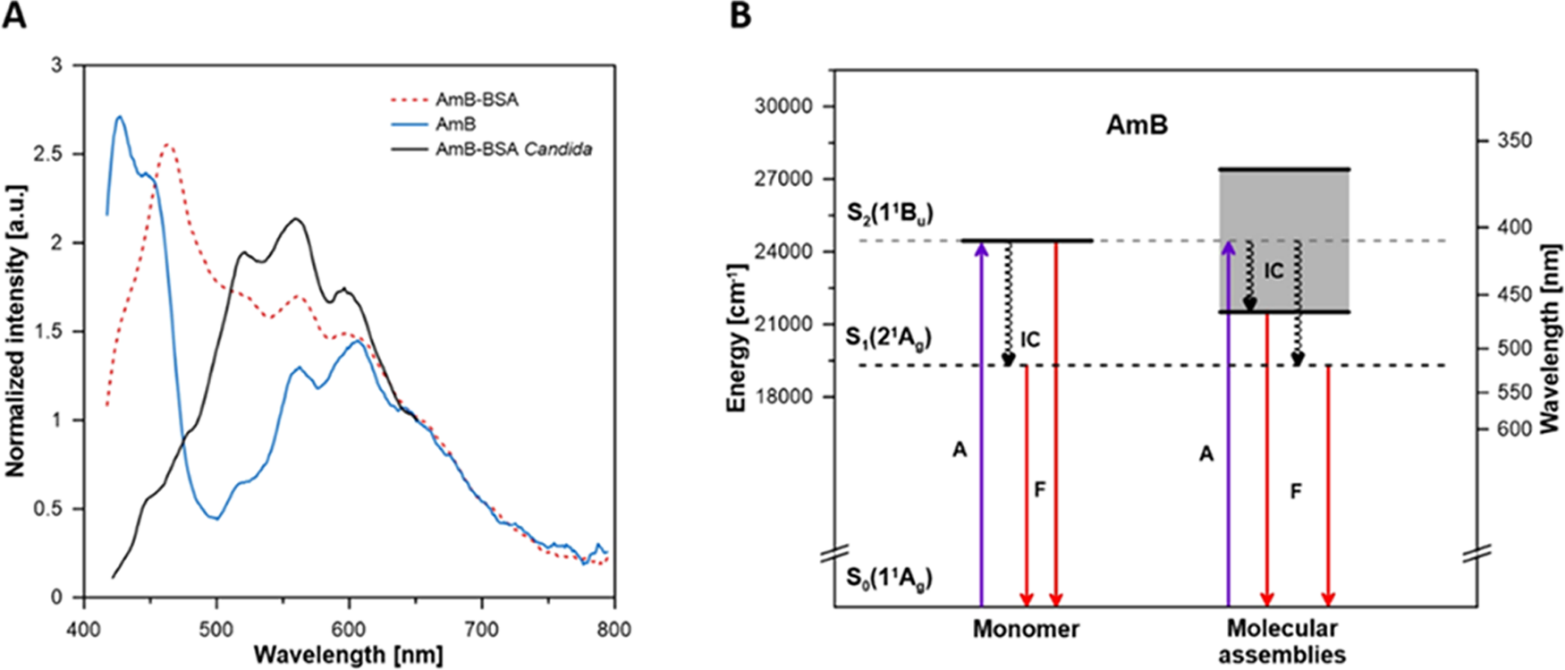
Fluorescence emission spectra of AmB in different systems (A) and interpretation of the electronic transitions observed (B). (A) Fluorescence emission spectra of the monomeric form of AmB in methanol (blue solid line), AmB–BSA complex in PBS buffer (red dashed line), and AmB–BSA complex incorporated into *C. albicans* cell membrane (back solid line) recorded directly from the single cell membrane under the microscope. (B) Energy-level diagram of AmB in the form of monomers and molecular assemblies, based on absorption and fluorescence spectra. Shaded areas represent excitonic bands. Note that in the case of AmB molecular assemblies, fluorescence emission is solely observed from the lowest excited singlet state (S_1_) owing to the efficient relaxation from the excitonic band.

**Figure 5:**
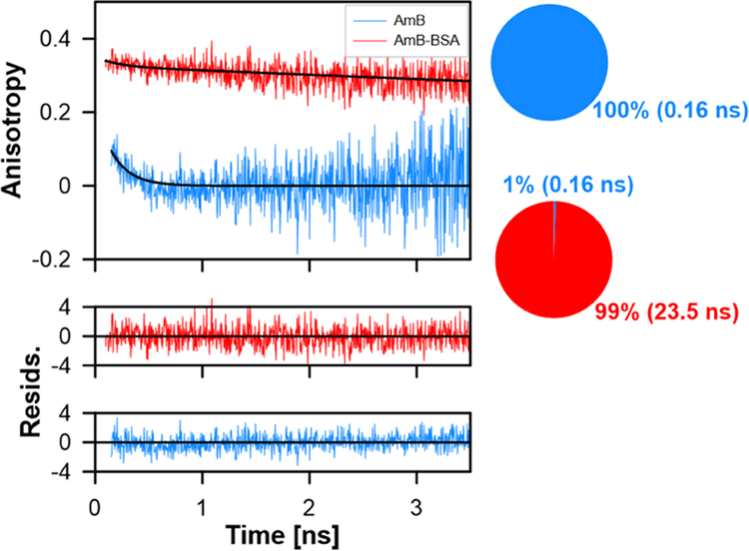
Anisotropy decays of AmB in methanol solution (blue line) and AmB–BSA complex in PBS buffer (red line). The decays represent rates of rotation of molecules (changes in the direction of AmB) in relation to the polarization direction of the excitation light. Rotational correlation times of free AmB (0.16 ns) and AmB bound to BSA (23.5 ns) have been determined based on fitting (black solid lines) to the decays. Percentage amplitudes of the rotational correlation times have been presented on circle diagrams. The bottom panels present the goodness of the fits.

The conclusion on the monomeric organization of AmB bound to BSA (single antibiotic molecules associated with the protein) has additional support from the analysis of the fluorescence emission spectra (Figure 4A).^21^ It has been demonstrated that monomeric AmB in solution gives rise to fluorescence emission characterized by two separate bands assigned to S_2_–S_0_ (400–500 nm) and S_1_–S_0_ (500–800 nm) transitions (see the energy diagram in Figure 4B).^21^ The emission spectrum recorded for AmB–BSA has also a two-band character, although the S_2_–S_0_ band shows a bathochromic shift (Figure 4A). The shift of the emission band in the short-wavelength region is most likely due to a solvatochromic effect resulting from the interaction of a single AmB molecule with the protein environment in the BSA binding site. An important issue is whether the drug molecules in AmB–BSA preparations exist only in the form of complexes or are additionally free in the water phase. To address this problem, we applied a rotational correlation time analysis (Figure 5).

Rotational diffusion of monomeric AmB in solution is characterized by a relatively short rotational correlation time of 0.16 ns.^22^ In contrast, the drug molecules immobilized in the AmB–BSA structures give rise to significantly longer correlation time values (23.5 ns). A component analysis shows that virtually all molecules of AmB in the preparation are present in the form of AmB–BSA complexes (99%, Figure 5). This result seems to be a direct consequence of the fact that AmB unbound to the protein and remaining in the aqueous phase was present in aggregated forms that were removed from the samples during centrifugation.

**Figure 6:**
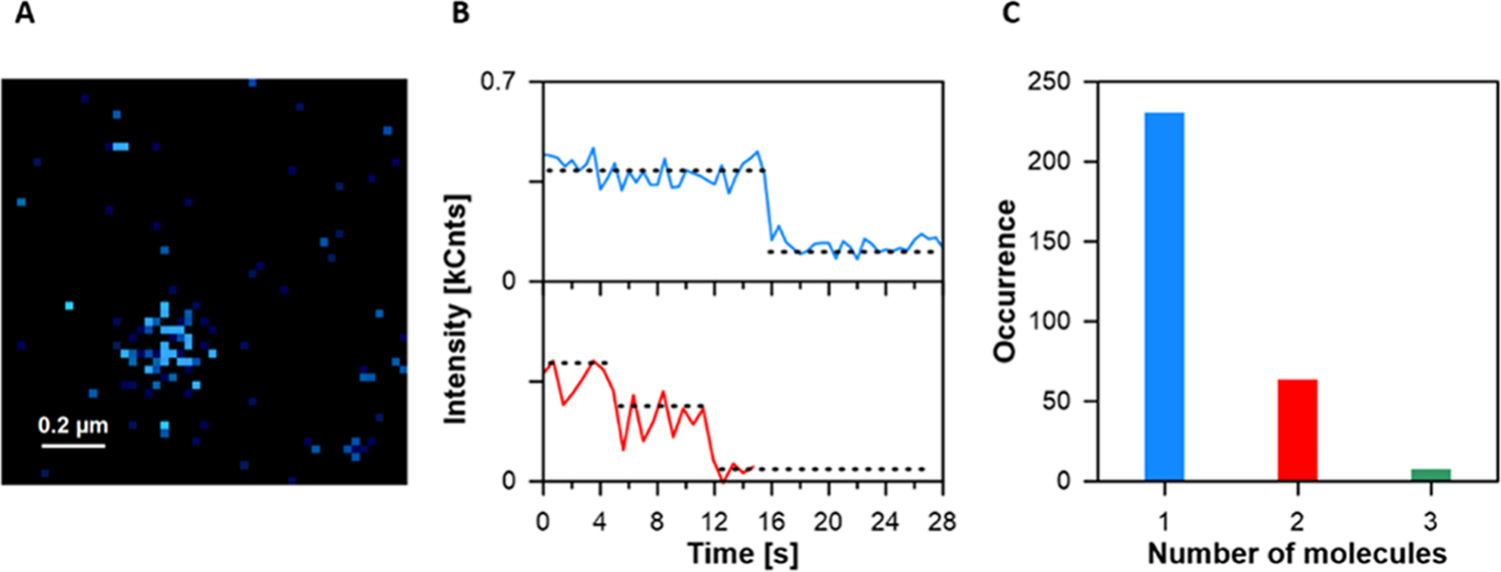
Single-particle fluorescence data of AmB–BSA complex. (A) Fluorescence intensity image of a single AmB–BSA particle deposited at the surface of the polylysine-coated slide. Additional exemplary images are shown in Figure S3. (B) Intensity–time traces characteristic for one (blue) and two (red) molecules of AmB complexed with BSA. The sudden drop in intensity represents the bleaching of one molecule. (C) Diagram representing the number of AmB molecules conjugated to single AmB–BSA particles, determined based on the intensity–time traces (such as those in panel B).

The problem of AmB and BSA stoichiometry in the formed complexes was also raised in experiments based on the analysis of single particles (Figure 6). Fluorescence microscopic analysis of the extremely diluted samples made it possible to detect light emission from single AmB–BSA particles (Figures 6A and S3). A prolonged illumination of a single particle (the timescale of seconds) results in a bleaching of the fluorophore and the number of bleaching steps directly reflects the number of fluorescent molecules in the particle in focus (Figure 6B). The analysis based on this methodology reveals that in most cases (>75%) a single BSA molecule hosts 1 molecule of AmB (Figure 6C), in accordance with the conclusions based on the results of the above-described experiments carried out with other techniques.

### Characterization of AmB–BSA Toxicity Against Red Blood Cells (RBC)

In order to examine the resistance of human membranes to the hemolytic agent, we suspended red blood cells in isotonic solutions with various addition and incubated these mixtures at 37 °C. One hour of incubation of erythrocytes in a solution containing 1 μM AmB did show a very small hemolytic effect equaling 3.7% only. However, incubation with BSA alone or AmB–BSA did not cause any hemolytic effect.

### Interaction of AmB–BSA and AmB Derivative Drugs with *C. albicans*

**Figure 7:**
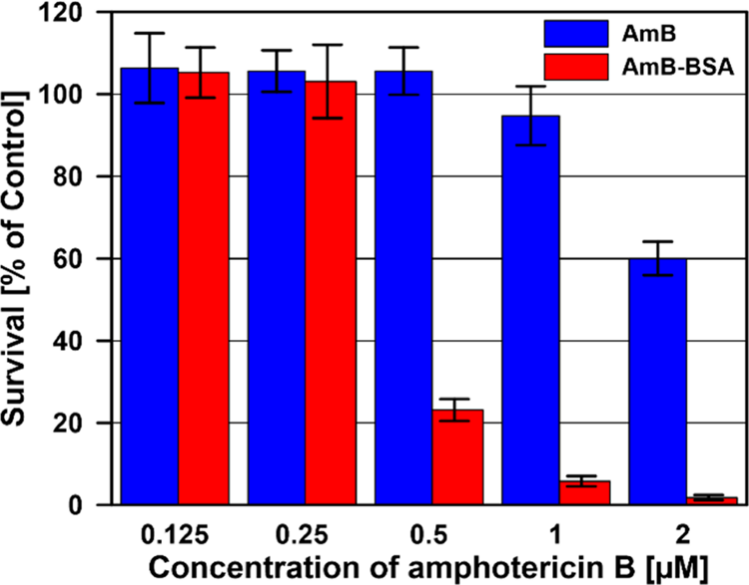
Comparison of the results of viability assays of *C. albicans* cells cultured in the presence of AmB and AmB–BSA complex. The results represent the arithmetic mean ± SD from three independent experiments performed with three repetitions for each type of sample. The control cells were incubated with 1% DMSO.

The antifungal efficacy of AmB–BSA against *C. albicans* was analyzed in a cell survival study (Figure 7). As can be seen, BSA-bound AmB exhibits a significantly enhanced antifungal efficacy far superior to that of pure AmB. Moreover, the activity of AmB–BSA is already observed at a relatively low concentration of the drug (<1 μM). BSA alone did not reduce the survival rate of *C. albicans* cells (Figure S4). The MIC and MFC values of AmB and AmB–BSA complex against *C. albicans* cells are presented in Table 1.

**Table 1:** Comparison of MIC and MFC Values of AmB Alone and AmB–BSA Complex toward *C. albicans* Cells

tested compound	MIC [μM]	MFC [μM]
AmB	0.78	6.25
AmB–BSA	0.125	0.5

We found it important and interesting to compare the antifungal activity of the AmB–BSA complex with the effect on the viability of fungal cells of the most popular and commonly used AmB preparations, i.e., AmBisome and Fungizone (Figure S5). As can be seen, both the formulations used at a concentration corresponding to that of AmB in the AmB–BSA complex (2 μM) did not decrease the viability of *C. albicans* cells after 0.5 h incubation. Even the 10 times higher dose of AmBisome (20 μM) did not inhibit the growth of fungal cells. Fungizone at the same concentration reduced survival of *C. albicans* cells by 81%.

**Figure 8:**
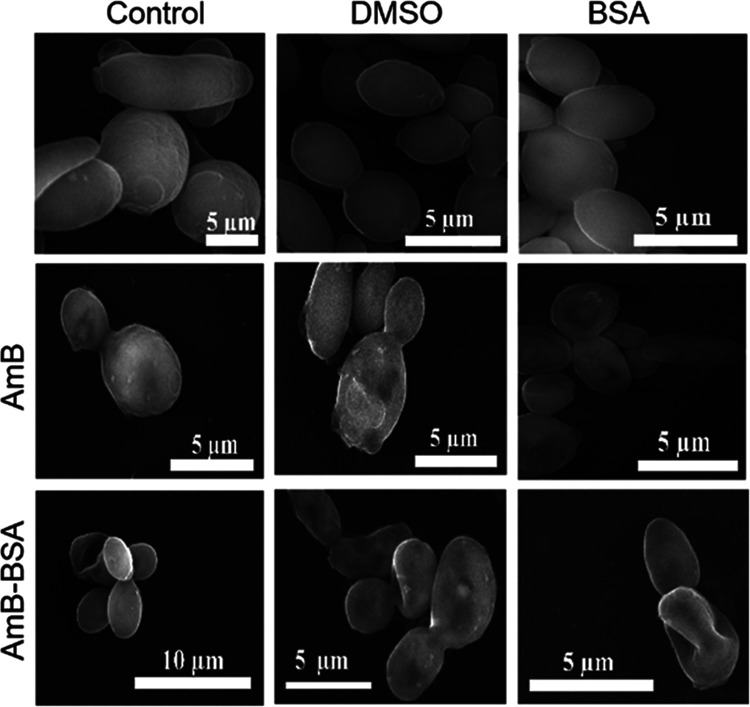
Scanning electron microscopy images of *C. albicans* cells. Control cells (upper row) were incubated with PBS (left hand), DMSO (middle), and with the addition of BSA (right hand) for 1 h. Middle row (all three pictures) images of cells from the culture incubated with AmB at a concentration of 2 μM. The lower row (all three pictures) shows cells from the culture incubated with AmB–BSA at the same concentration of the drug.

In order to further analyze the high effectiveness of AmB–BSA against *C. albicans,* the topography of the cells was examined with the application of scanning electron microscopy (SEM). The cells were incubated for 1 h (Figure 8) and 2 h (Figure S6) with DMSO, BSA, AmB, and AmB–BSA. The three different control cells, incubated with PBS, 1% DMSO, or BSA revealed typical *C. albicans* morphology. Most cells were spherical or ovoid-shaped and on the smooth surface, there were visible buds and scars positioned at the tips of cells. Scars were found in multiple (mostly in two) or singular forms and were located mainly at the poles. Occasionally, extended forms of the cells were also found (Figures 8 and S6). Tiny changes were noted under the influence of AmB at the concentration applied in this test. Most of the cells were not changed. In others, with morphological alteration, very small indentations or shady zones of future indentations have been found. Conversely, significant changes in morphology were revealed in the cells treated with AmB–BSA at the drug concentration of 2 μM. Some cells were elongated with collapsed walls. Characteristic indentations, even from both sides, were well discernible. Other cells have altered shape and some shrinkage was observed. When assessing the changes in morphology, one should consider MIC and MFC data that are coupled to them. It was shown that at both tests, the complex was more efficient in inhibitory and fungicidal action. SEM analysis of the changes in the morphology of *C. albicans* cells after 1 h incubation with 2 μM concentration of pure AmB or AmB–BSA complex were observed in 9 and 19% of cells, respectively. For 2 h of incubations, it was 18 and 30%, for AmB and AmB–BSA complex, respectively. The SEM observation made in the current study provides evidence for stronger fungicidal action exerted by AmB complexed with BSA as compared to AmB itself at the same examined dose. The deformed phenotypes of the cells, observed in this investigation, strongly support such a conclusion. Analogical studies have been performed in order to analyze the effectiveness of AmBisome and Fungizone against *C. albicans*. The topography of the cells was examined with the application of SEM (Figure S7). The cells were incubated for 1 and 2 h with buffers (controls) and with drugs at the final AmB concentration of 2 μM. In all of the experimental variants, typical morphology has been revealed. Most cells were spherical or ovoid-shaped and on the smooth surface, there were visible buds and scars positioned at the tips of cells. Any changes were noted under the influence of AmBisome and Fungizone at any time at the concentration applied in this investigation.

**Figure 9:**
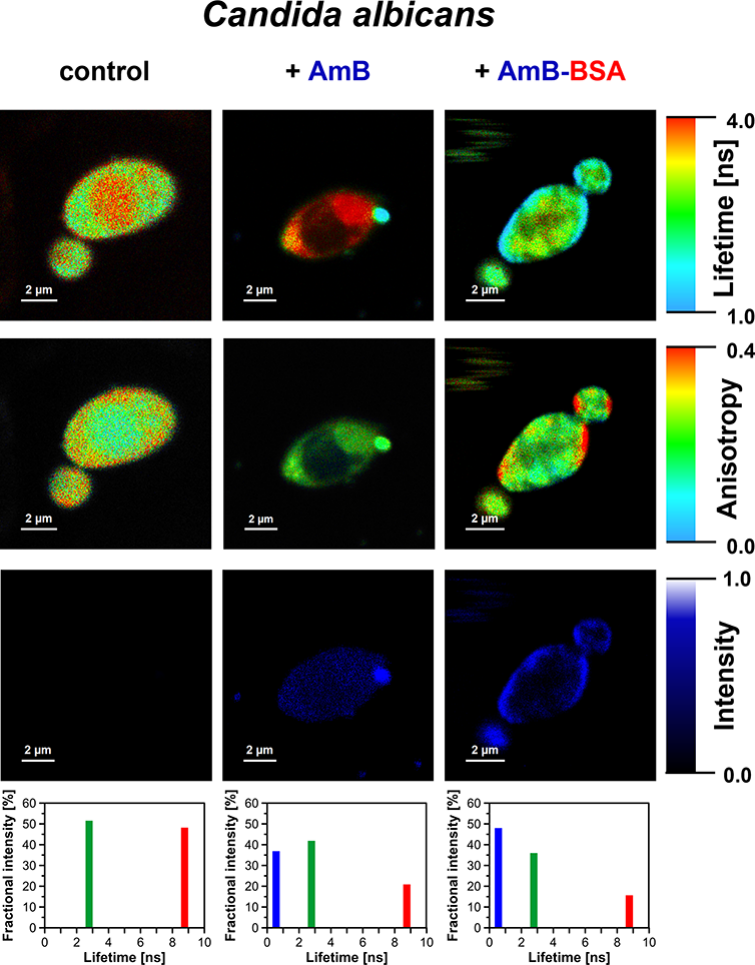
Image of *C. albicans* cells before (left panels) and after exposure of the cells to AmB (middle panels) and AmB–BSA (right panels). AmB concentration of 2 μM. The upper row presents images based on fluorescence lifetime, in the second row the same cells are shown imaged based on fluorescence anisotropy values, and the third row shows the images based on an amplitude of the short-lifetime fluorescence component (0.56 ns) assigned to AmB. At the bottom, the fluorescence lifetime analysis of the cells presented in the upper panels is shown. The following fluorescence lifetime components were resolved: 2.8 ns (green) and 8.8 ns (red) assigned to the cell autofluorescence and 0.56 ns (blue) assigned to AmB. The data has been gathered with excitation laser light at 405 nm.

A very important problem regarding the molecular mechanism underlying the exceptionally high antifungal activity of BSA-bound AmB can be attempted to be solved with the application of fluorescence lifetime imaging microscopy (FLIM). Figure 9 shows FLIM images of *C. albicans* cells exposed to AmB–BSA. Two fluorescence lifetime components can be resolved in the endogenous emission of the cells: 2.8 and 8.8 ns (Figure 9, bottom panel). An additional, relatively short fluorescence lifetime component can be resolved in the cells exposed to AmB or AmB–BSA (0.56 ns) assigned to AmB in the form of small aggregated structures formed in the natural environment of both human and fungal cells.^13,14,21^ As can be seen from Figure 9, such structures (represented by blue color code) are located predominantly in the cell membranes, although penetration of AmB into the cytoplasm also can be observed, in particular, in the case of young cells (see also Figure S8). The conclusion regarding the presence of AmB in the form of small molecular aggregates in cell membranes is additionally supported by the analysis of fluorescence emission spectra recorded locally by means of emission microspectroscopy with detection focused on a single cell membrane (Figure 4A). The fact that the emission spectrum recorded from a single cell membrane of *C. albicans* consists of a single band in the long-wavelength region is an expression of the efficient internal energy conversion from the S_2_ to S_1_ state associated with the presence of an excitonic band characteristic of molecular assemblies (Figure 4A, black solid line). The relatively high fluorescence intensity of AmB in the cell membranes located in the left-hand and right-hand sectors of the cell (Figure 9 third row and Figure S8), combined with the higher fluorescence anisotropy values in the same regions (Figure 9 second row), is a manifestation of the orientation of AmB molecules in the membrane perpendicular to the lipid bilayer plane, as can be deduced based on the photoselection studies.^23^ The same conclusion regarding the orientation of AmB in biomembranes has been reached recently based on the photoselection studies carried out with the application of fluorescence^13,14^ and Raman imaging^24^ analyses.

Importantly, the fact that AmB molecules that entered the *C. albicans* cells are present as aggregated structures, both in the membranes and in the cytoplasm, implies that the mode of the biological activity of this antibiotic is associated with its molecular organization. One of the structures of AmB proposed to be responsible for the fungicidal activity of this antibiotic is a transmembrane pore affecting physiological ion transport.^5,25^ As can be seen, aggregated AmB structures (appearing in blue in FLIM images) are mainly present in cell membranes and newly formed cell buds (Figures 9 and S8). Based on this observation, we found in our previous studies that the cell wall protects cells from AmB incorporation into lipid membranes by trapping antibiotic molecules. As can be seen from the comparison of the mode of action of pure AmB and AmB transported by BSA (Figure 9), AmB–BSA complexes are much more effective in crossing the cell wall barrier and delivering the antibiotic to the cell membrane, which is the target site of its biological activity. Most probably, this is responsible for the exceptionally high antifungal activity of AmB–BSA as compared to AmB present in the water phase (Figure 7) or in the form of pharmacological preparations such as Fungizone and AmBisome (Figure S5). As can be seen from the FLIM images of *C. albicans* cells exposed to AmBisome and Fungizone (Figures S9 and S10), the distribution of AmB in the cells and the efficiency of incorporation of the antibiotic delivered by these preparations resembles pure AmB rather than complexed with protein in AmB–BSA.

## Conclusions

**Figure 10:**
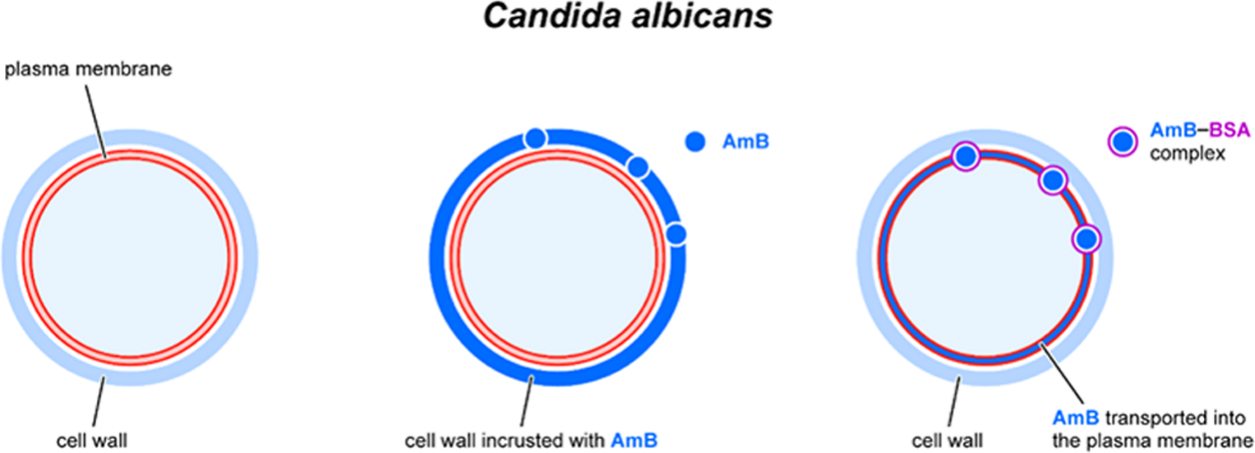
Schematic representation of binding of AmB and AmB–BSA complex to a fungal cell. The model is discussed in the text.

Regardless of the exact molecular mechanism underlying the biological activity of AmB, the drug is recognized as a highly effective and life-saving antibiotic. The results of detailed studies based on molecular spectroscopy and imaging^13^ show that the binding of AmB molecules to the cell wall structures of fungal cells is a kind of “self-defense” of the cells against the toxicity of the drug, which can significantly affect its therapeutic effectiveness (see the diagram in Figure 10).

Here, we show that this line of defense can be overcome by using an antibiotic carrier, such as a protein capable of binding AmB in the aqueous phase, crossing the cell wall barrier and rereleasing the drug into the lipid membrane environment. In our opinion, such a mechanism facilitating the transfer of AmB molecules to fungal cells is responsible for the exceptionally high antifungal effect of this antibiotic (Figures 7 and 9) and can be called the ″Trojan Horse″ effect of the protein carrier. Importantly, the present study showed that the AmB–BSA system had no hemolytic effect on human red blood cells. The FLIM imaging results show that AmB molecules transferred to fungal cells are mainly located in cell membranes, are oriented perpendicular to the membrane plane, and form molecular aggregates (Figures 9 and S8). Such aggregated structures may likely act as transmembrane pores, disrupting the physiological ionic balance, thus leading to cell death.^5^ A comparison of the antifungal activity of the AmB complex with BSA and in other systems commonly used in the treatment of fungal infections (Figures 7 and S5) shows that AmB–BSA can be effectively used at much lower concentrations of the antibiotic, which implies less toxicity to human cells.^14^ Since animal cells do not have a cell wall as a protective barrier against AmB entry into their membranes, fungal cells are in a privileged position to avoid the toxicity of this antibiotic. AmB in the form of complexes with protein carriers can effectively cross the barrier of the fungal cell wall increases, in our opinion, the potential therapeutic value of the antibiotic.

## Supporting Information Available

Particle size distribution of BSA and AmB–BSA complex solutions (Figure S1), FTIR spectra of AmB–BSA (Figure S2), fluorescence intensity images of single AmB–BSA particles (Figure S3), diagram of survival of *C. albicans* cells incubated with different concentrations of BSA (Figure S4), comparison of the results of viability assays of *C. albicans* cells cultured in the presence of Fungizone and AmBisome (Figure S5), additional SEM images of *C. albicans* cells incubated with AmB and AmB–BSA complex for 2 h (Figure S6), additional SEM images of *C. albicans* cells incubated with AmBisome and Fungizone (Figure S7), images of *C. albicans* cells based on an amplitude of the fluorescence lifetime component assigned to AmB (Figure S8), time-resolved and anisotropy fluorescence images of *C. albicans* after exposition to AmBisome (Figure S9), and time-resolved and anisotropy fluorescence images of *C. albicans* after exposition to Fungizone (Figure S10) (PDF)
