# Supplementary material for: Enhanced Antifungal Activity of Amphotericin B Bound to Albumin: A “Trojan Horse” Effect of the Protein
Source: J Phys Chem B. 2023 Apr 18;127(16):3632–40. doi: 10.1021/acs.jpcb.3c01168 (PMC10150355; doi:10.1021/acs.jpcb.3c01168)
Supplement: Supplementary file 1 — jp3c01168_si_001.pdf [file jp3c01168_si_001.pdf]

Supporting Information

Enhanced Antifungal Activity of Amphotericin B Bound to Albumin: A “Trojan Horse” Effect of the Protein

*Ewa Grela[1], Sylwia Stączek[2], Monika Nowak[1], Bozena Pawlikowska-Pawlega[3], Agnieszka Zdybicka-Barabas[2], Sebastian Janik[1], Małgorzata Cytryńska[2], Wojciech Grudzinski[1], Wieslaw I. Gruszecki[1], Rafal Luchowski[1] **

[1]Department of Biophysics, Institute of Physics, Faculty of Mathematics, Physics and Informatics, Maria Curie-Sklodowska University, 20-031 Lublin, Poland

[2]Department of Immunobiology, Faculty of Biology and Biotechnology, Institute of Biological Sciences, Maria Curie-Sklodowska University, 20-033 Lublin, Poland

[3]Department of Functional Anatomy and Cytobiology, Faculty of Biology and Biotechnology, Institute of Biological Sciences, Maria Curie-Sklodowska University, 20-033 Lublin, Poland.

*Corresponding author: Rafal Luchowski, e-mail: rafal.luchowski@umcs.pl, tel.: +4881-537-62-47, fax: +4881-537-6191

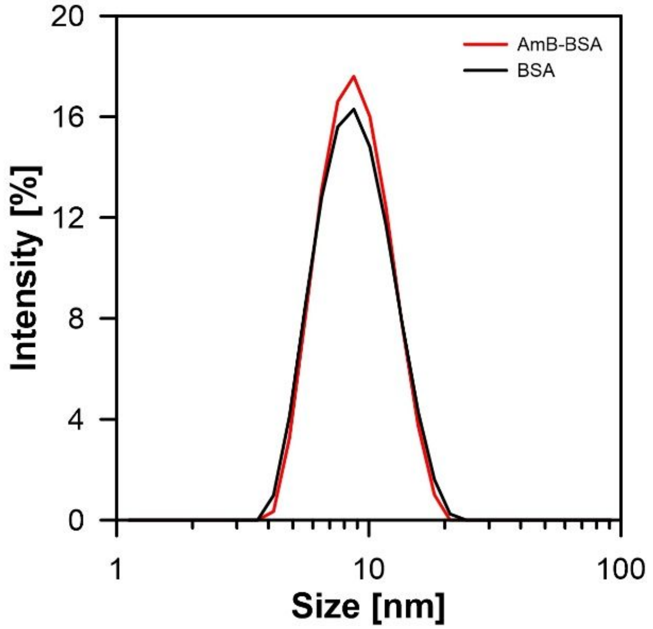

Figure S1. Particle size distribution determined with the application of Zeta-sizer in a solution of BSA and AmB-BSA (marked).

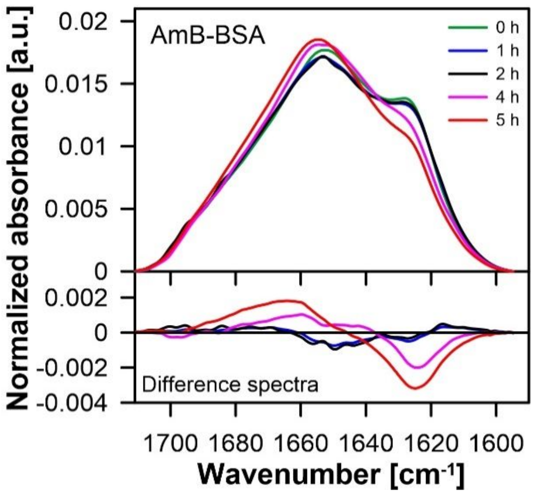

Figure S2. FTIR spectra of the AmB-BSA complex, in the amide I region, recorded immediately after the preparation (time 0) and after different time periods indicated. Lower panel presents the difference spectra calculated based on the spectra shown in the upper panel: the initial spectrum minus the spectrum recorded after different incubation periods (marked by the same color codes).

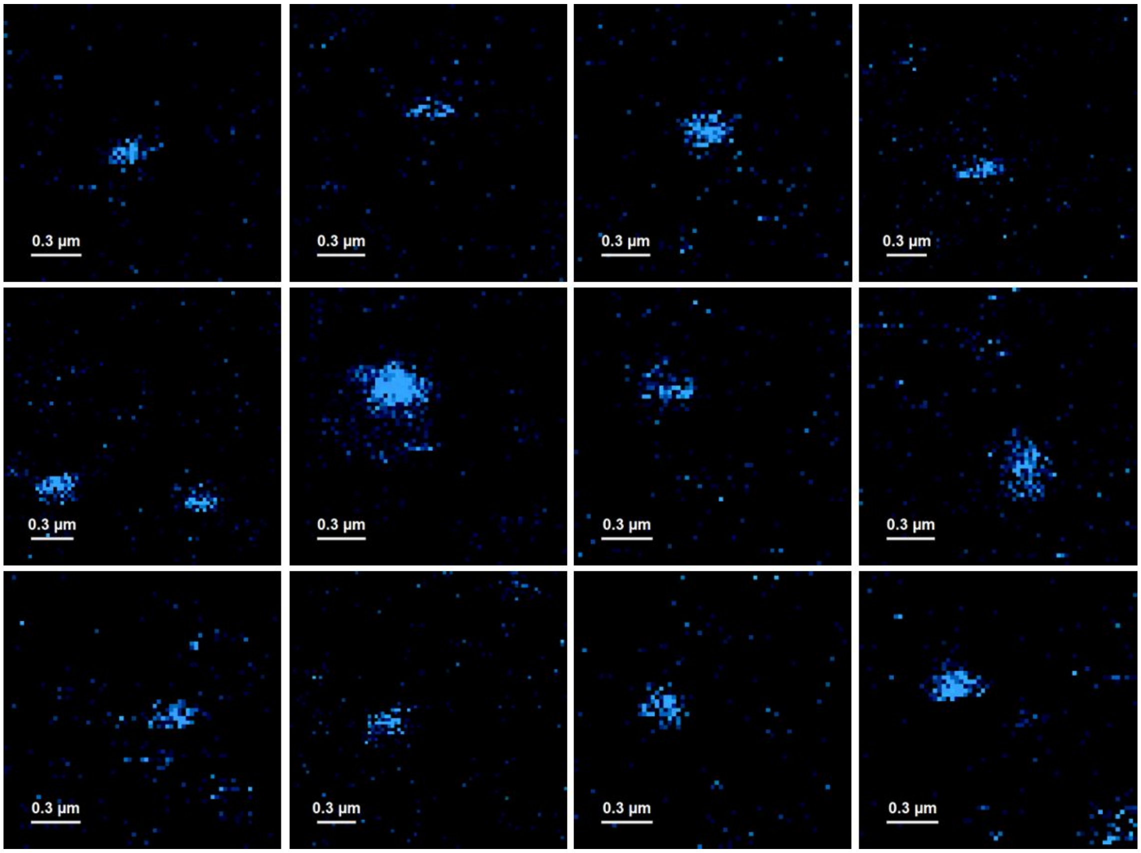

Figure S3. Fluorescence intensity images of single AmB-BSA particles deposited at the surface of the polylysine-coated slides.

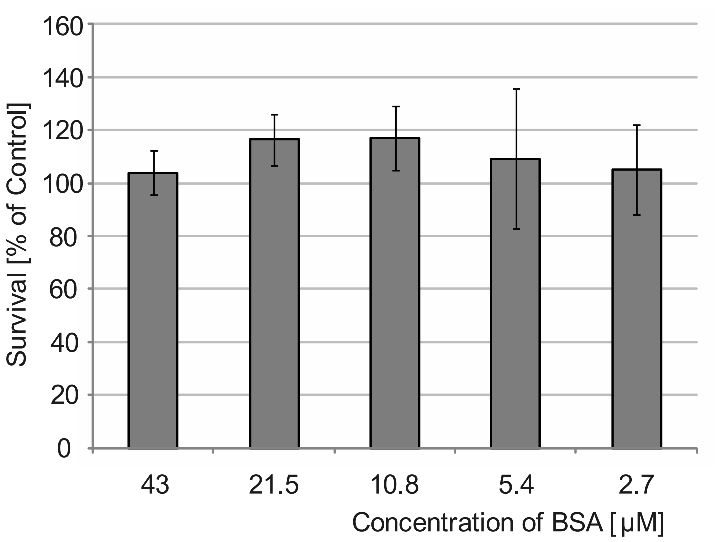

Figure S4. Survival of *C. albicans* cells incubated with different concentrations of BSA corresponding to the protein levels in the tested AmB-BSA complexes. Control cells were incubated with 1% of DMSO.

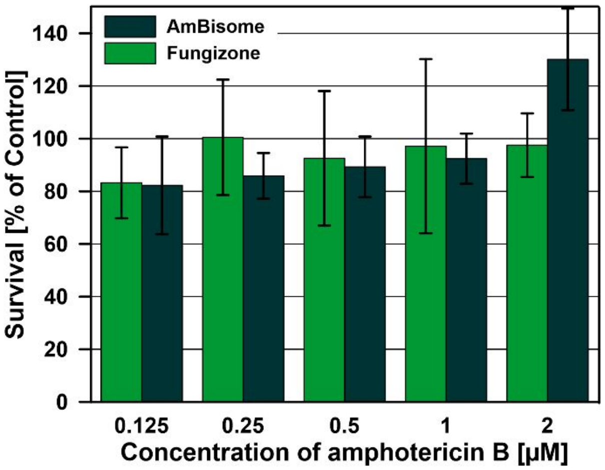

Figure S5. Comparison of the results of viability assays of *C. albicans* cells cultured in the presence of Fungizone and AmBisome. The results represent the arithmetic mean ± S.D. from three independent experiments performed with three repetitions for each type of sample. The control cells were incubated with the buffer containing disodium succinate hexahydrate and sucrose or PBS for Ambisome or Fungizone, respectively.

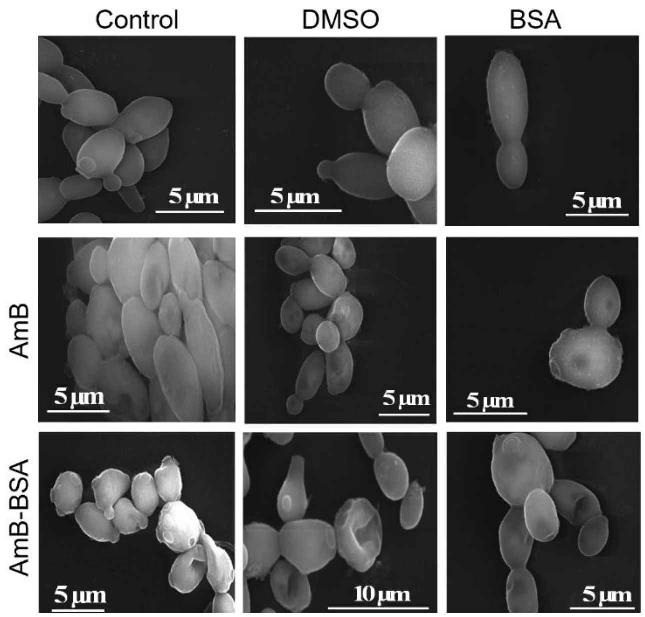

Figure S6. Scanning Electron Microscopy images of *C. albicans* cells. Control cells (upper panel) were incubated with PBS (left hand), DMSO (middle) and with the addition of BSA (right-hand) for 2 hours. Middle panel (three pictures) images of cells from the culture incubated with AmB at the concentration of 2 µM. Lower panel (three images) shows cells from the culture incubated with AmB-BSA at the same concentration of the drug.

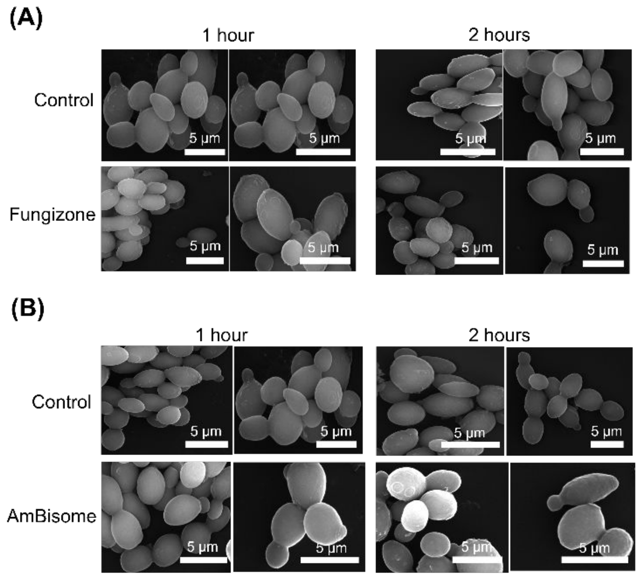

Figure S7. Scanning Electron Microscopy images of *C. albicans* cells. (A) Control cells (upper row), cells incubated with Fungizone (bottom row). (B) control cells (upper row), cells incubated with AmBisome (bottom row). The cells were incubated for 1 and 2 hours at final dose of 2 μM of AmB.

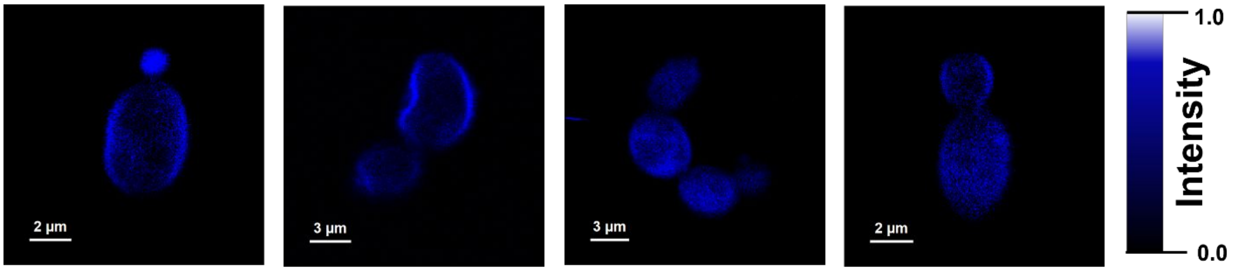

Figure S8. Images of *C. albicans* cells based on an amplitude of the short-lifetime fluorescence component (0.56 ns) assigned to AmB. See also Fig. 9. Before imaging cells were exposed to AmB-BSA at an AmB concentration of 2 μM.

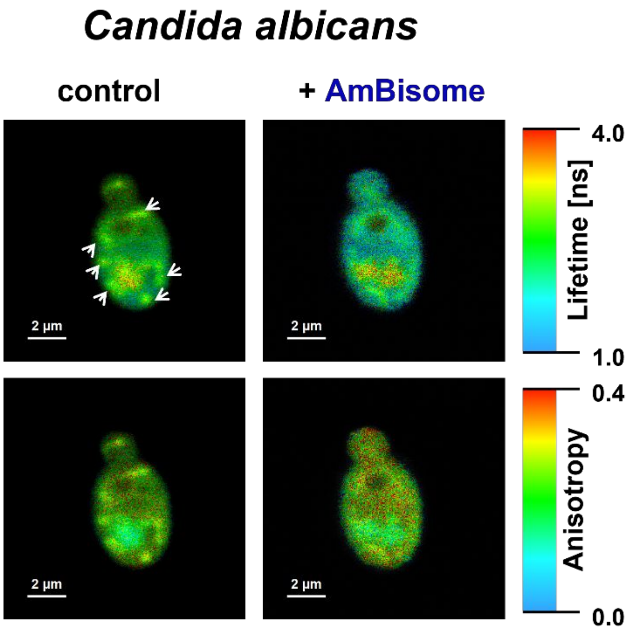

Figure S9. Images of *C. albicans* cells before (left panels) and after (right panels) exposition of the cells to AmBisome. Upper panels present images based on fluorescence lifetime, below, the same cells are shown imaged based on fluorescence anisotropy values. Blue color code in the FLIM image represents an amplitude of the short-lifetime fluorescence component (below 1 ns) assigned to AmB and the following fluorescence lifetime components 2.8 ns (green) and 8.8 ns (red) are assigned to the cell autofluorescence. Organelles are pointed by white arrows.

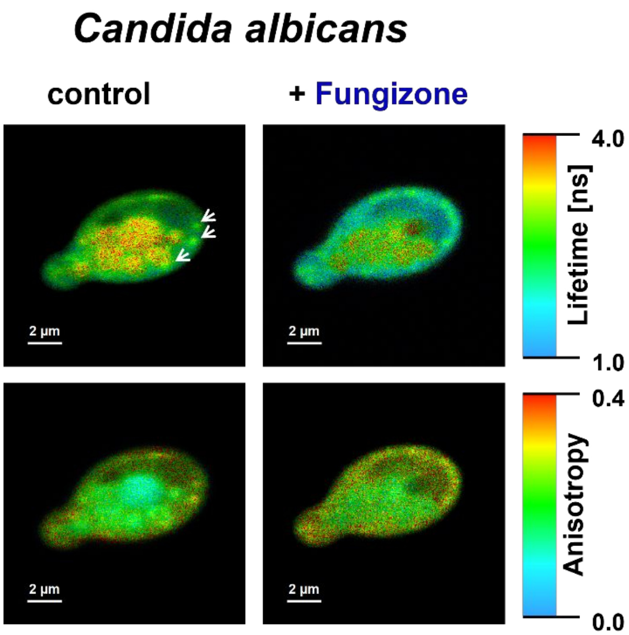

Figure S10. Images of *C. albicans* cells before (left panels) and after (right panels) exposition of the cells to Fungizone. Upper panels present images based on fluorescence lifetime, below, the same cells are shown imaged based on fluorescence anisotropy values. Upper panels present images based on fluorescence lifetime, below, the same cells are shown imaged based on fluorescence anisotropy values. Blue color code in the FLIM image represents an amplitude of the short-lifetime fluorescence component (below 1 ns) assigned to AmB and the following fluorescence lifetime components 2.8 ns (green) and 8.8 ns (red) are assigned to the cell autofluorescence. Organelles are pointed by white arrows.
